# Meeting Report: Alternatives for Developmental Neurotoxicity Testing

**DOI:** 10.1289/ehp.9841

**Published:** 2007-01-29

**Authors:** Pamela Lein, Paul Locke, Alan Goldberg

**Affiliations:** 1 Department of Environmental Health Sciences and Center for Alternatives to Animal Testing (CAAT), Johns Hopkins University Bloomberg School of Public Health, Baltimore, Maryland, USA; 2 Center for Research on Occupational and Environmental Toxicology, Oregon Health & Science University, Portland, Oregon, USA

**Keywords:** alternatives, developmental neurotoxicity testing, humane science, *in vitro* models, policy, regulatory acceptance, screening, TestSmart, the 3Rs, validation

## Abstract

Developmental neurotoxicity testing (DNT) is perceived by many stakeholders to be an area in critical need of alternatives to current animal testing protocols and guidelines. To address this need, the Johns Hopkins Center for Alternatives to Animal Testing (CAAT), the U.S. Environmental Protection Agency, and the National Toxicology Program are collaborating in a program called TestSmart DNT, the goals of which are to: (*a*) develop alternative methodologies for identifying and prioritizing chemicals and exposures that may cause developmental neurotoxicity in humans; (*b*) develop the policies for incorporating DNT alternatives into regulatory decision making; and (*c*) identify opportunities for reducing, refining, or replacing the use of animals in DNT. The first TestSmart DNT workshop was an open registration meeting held 13–15 March 2006 in Reston, Virginia. The primary objective was to bring together stakeholders (test developers, test users, regulators, and advocates for children’s health, animal welfare, and environmental health) and individuals representing diverse disciplines (developmental neurobiology, toxicology, policy, and regulatory science) from around the world to share information and concerns relating to the science and policy of DNT. Individual presentations are available at the CAAT TestSmart website. This report provides a synthesis of workgroup discussions and recommendations for future directions and priorities, which include initiating a systematic evaluation of alternative models and technologies, developing a framework for the creation of an open database to catalog DNT data, and devising a strategy for harmonizing the validation process across international jurisdictional borders.

## Background

There is considerable concern that chemical exposures are contributing to the increasing incidence of neurodevelopmental diseases in children (Schettler 2001). However, most chemicals have not been evaluated for their potential to cause developmental neurotoxicity (Goldman and Koduru 2000). To fill this information gap, industry is increasingly being asked to provide adequate information for the assessment of developmental neurotoxicity in its products and wastes. In response, developmental neurotoxicity testing (DNT) needs are expected to increase significantly in the United States and globally. Current DNT testing guidelines, U.S. Environmental Protection Agency (EPA) 870.6300 DNT Guideline (U.S. EPA 1998) and the draft OECD 426 [Organisation for Economic Co-operation and Development (OECD) 2006] are expensive in terms of scientific resources, time, money, and animals (Lein et al. 2005), and risk-based criteria for setting testing priorities have yet to be developed. Relying solely on the existing guidelines to address current and anticipated future regulatory demands for DNT of the thousands of chemicals for which there is little to no DNT data would incur unacceptable costs in terms of animals and person-years. There is also increasing pressure on the scientific and regulatory communities from environmental health and children’s welfare advocacy groups to test more chemicals for their potential to harm the developing human nervous system. Thus, there is strong incentive to create alternative methodologies that meet the expressed demands for identifying potential developmental neurotoxic agents with speed, reliability, and respect for animal welfare.

The Johns Hopkins Center for Alternatives to Animal Testing (CAAT) introduced its first TestSmart program in 1999 to develop a more humane and efficient approach to risk assessment of high production volume (HPV) chemicals. The TestSmart process brings together diverse stakeholder groups from around the world, including research scientists, government scientists, regulators, policy analysts, industry representatives, academics, and public interest groups, encouraging them to work collaboratively so that as the science is developed, its policy implications will be understood, and, as policy needs are articulated, the science can respond. After the TestSmart HPV workshop (26–27 April 1999, Fairfax, VA, the U.S. EPA announced it had decreased its estimates for animal use by 80%. This estimate was based partly on the TestSmart HPV recommendation that the OECD combine required assays and, where possible, replace *in vivo* tests with *in vitro* OECD assays. These data are available from this website: http://caat.jhsph.edu/programs/workshops/testsmart/index.htm (CAAT 2007). Building on the success of this program, CAAT is collaborating with the U.S. EPA and the National Toxicology Program (NTP) to adapt the TestSmart approach to DNT. The goals of TestSmart DNT are to *a*) identify alternative DNT models based on evolutionarily conserved neurodevelopmental end points of mechanistic relevance to developmental neurotoxicity; *b*) develop the process for validating candidate alternative methods both scientifically and in terms of regulatory acceptance; and *c*) identify opportunities for reducing, refining, or replacing the use of animals in DNT. These goals were derived in part from a workshop sponsored by the CAAT, the European Chemical Industry Council (Cefic), and the European Center for the Validation of Alternative Methods (ECVAM) in April 2005 in Ispra, Italy, which examined the current status of alternatives for DNT testing. A report of this workshop (Coecke et al. 2007) has been published online by *Environmental Health Perspectives*.

## Workshop Objectives

The primary objective of the first international meeting of TestSmart DNT was to bring together the various stakeholders (test developers, test users, regulators and advocates for children’s health, animal welfare and environmental health) and individuals representing diverse disciplines (developmental neurobiology, toxicology, policy and regulatory science) to exchange information and concerns relating to DNT science and policy. Additionally, the workshop sought to: (*a*) outline the opportunities for and challenges of DNT alternatives; (*b*) initiate dialogue on policy requirements for validating candidate alternatives; (*c*) discuss the 3Rs (refining, reducing, and replacing the use of animals) as they apply to DNT; (*d*) establish a community of scientists and policy makers who will work together to integrate and coordinate the science and policy of alternative DNT as it evolves; and (*e*) set the agenda for the second TestSmart DNT meeting (12–14 November 2006, Reston, VA).

## Workshop Summary: Challenges and Opportunities

### The science of DNT

#### Strategic approach to developing DNT alternatives

A fundamental scientific principle that has driven the successful development of alternatives for toxicity testing in organ systems is that highly conserved events in the biology of a system or a process provide a robust source of new efficient methods in target organ testing (Lein et al. 2005). For example, the predictive power of *in vitro* mutagenesis tests as screens for genotoxic agents results from their incorporation of a relevant biologic mechanism (DNA mutation) that is conserved across species. Applying this principle to developing alternatives for DNT is challenging because the molecular mechanism(s) by which developmental neurotoxicants perturb neurodevelopment are not well characterized. Moreover, because of the complexity of the developing brain, it is likely that there are many molecular mechanisms of developmental neurotoxicity, a conclusion borne out by mechanistic studies of neurodevelopmental diseases. However, significant advances in our understanding of the cellular and molecular mechanisms of neurodevelopment over the past 10 years have identified and characterized key cellular events that are critical to the formation of a functional nervous system. These include neural induction, precursor cell proliferation, pattern formation, cell migration, neuronal and glial differentiation, formation of axons and dendrites, axonal guidance and target recognition, cell survival and apoptosis, synapse formation and pruning, and neurotransmitter specification. It is now clear that the fundamental principles underlying these cellular events of neurodevelopment are remarkably conserved across species ranging from the nematode (*Caenorhabditis elegans*) to human. These advances provide the opportunity to develop DNT alternatives focused on end points that capture key evolutionarily conserved neurodevelopmental events. This strategy is based on the prevailing thought in the field that many developmental neurotoxicants, regardless of their actual mechanism of action, share common final outcomes of altered cell proliferation and survival, aberrant cell differentiation, or disrupted neuronal connectivity (Barone et al. 2000; Lein et al. 2005; Slotkin 2004). The relevance of these end points to developmental neurotoxicity is further supported by clinical studies of children and experimental studies in primates, rodents, and simpler organisms, indicating that perturbation of these key neurodevelopmental events underlies functional deficits associated with neurodevelopmental diseases of environmental and/or genetic origin (Rice and Barone 2000).

#### *In vitro* models and DNT

*In vitro* cell-based models that recapitulate each of the neurodevelopmental events listed above have been developed to examine cellular and molecular mechanisms of neurodevelopment. These models, listed in order of increasing complexity, include neurotypic and gliotypic cell lines, primary neuronal and/or glial cell cultures, reaggregate brain cultures, brain slices, and organotypic explants. End points that can be measured in these models include cellular morphology, biochemical markers, neuro-transmission, and molecular events (e.g., gene expression and intracellular signaling). There is considerable ongoing effort to develop the technology for high(er) throughput/high(er) content analyses of these end points. More complex *in vitro* models, such as brain slices, have also been adapted to study functional correlates of complex behaviors, such as long-term potentiation and synaptic plasticity. Most of the primary cell culture models use tissues from avian or rodent sources; however, of particular interest to DNT is the availability of human embryonic stem cells for culture. Human neural progenitor cells, human stem cell lines, and human cord blood–derived stem cell models recapitulate several key neurodevelopmental events, including differentiation of precursor cells into neurons and glia and the formation of neural networks. These models could potentially reduce uncertainty in cross-species extrapolation.

The major advantage of *in vitro* cell-based models is the ability to replicate discrete stages of neurodevelopment in a relatively simple system amenable to monitoring and manipulation of gene expression. Because these models reduce the complexity of the developing nervous system, it is easier to detect subtle but functionally important changes in cell proliferation and differentiation and neuronal connectivity that are not easily observed in rodent models. The availability of technology to monitor or experimentally manipulate gene expression provides the opportunity to integrate molecular data with structural and functional observations at the cellular level and allows adaptation of models to incorporate genetic polymorphisms known or suspected to influence human susceptibility to developmental neurotoxicants. However, adapting *in vitro* cell-based models for DNT also poses significant challenges. Because developmental neurotoxicity is contextually driven—neurotoxic effects vary depending on brain region, developmental stage, and genetic background—the use of these models raises concerns about missing critical cell–cell or gene–environment interactions as well as differences between brain regions and between developmental stages (Lein et al. 2005). Another limitation of *in vitro* cell-based models is the lack of integrated systemic functions, which increases the probability of missing toxicant effects on extra-neural factors that influence neurodevelopment, such as metabolism, immune mediators, and endocrine function.

#### Alternative systems-based models and DNT

The complexity of interdependent events in the developing nervous system raises concerns as to whether a DNT test battery based on a few well-defined end points reproduced *in vitro* using cell-based models will be predictive of *in vivo* effects. Therefore, systems-based models employing simple organisms that enable assessment of integrative effects may offer a more immediately viable and ultimately more powerful approach to DNT alternatives. Simple organisms that have been proposed as candidate DNT alternatives include (listed in order of increasing evolutionary complexity) sea urchins, nematodes (*C. elegans*), flies (*Drosophila melanogaster*), fish [zebrafish (*Danio rerio*) and medaka (*Oryzias latipes*)], and chick embryos. Of these, *C. elegans*, *Drosophila*, zebrafish, and chick embryos have been used most extensively in mechanistic studies of developmental neurobiology and there exists a significant knowledge base regarding cellular and molecular regulation of neurodevelopment in these organisms. Such studies demonstrate that although there are notable differences between neurodevelopment in these simpler organisms and mammals, the fundamental processes of neurodevelopment are analogous to those that occur in humans. Moreover, genes homologous to human genes linked to neurodevelopmental diseases are expressed in these models. The complete genome has been sequenced for *C. elegans*, *Drosophila,* zebrafish, and medaka, the technology is in place to monitor and experimentally manipulate gene expression in nematodes, flies, fish, and chick embryos, and some behavioral tests have been developed for these models. Thus, tools are available to integrate developmental neurotoxicity studies at the biochemical, molecular, and cellular level with alterations in structure, function, and behavior. This will allow direct testing of cause–effect relationships between toxicant effects on biochemical, cellular, or molecular end points and effects on apical measures such as behavior. In addition, this information could be used to construct a genomics/proteomics database for predicting developmental neurotoxic potential across species at the most fundamental level of homology. Ongoing studies are focused on developing the technology to adapt these systems-based *in vivo* models for high(er) throughput/high(er) content analyses of genomic, proteomic, structural, biochemical, and behavioral end points.

A significant advantage of all these organisms is their small size, rapid embryonic development, and short life cycle, which will greatly reduce time and space costs. Zebrafish and medaka offer an advantage relative to the other models in that their embryos are optically transparent. Thus, it is possible to visualize dynamic changes in gene expression (using fluorescent reporter transgenic lines) and detailed morphogenetic movements as they occur in the live developing embryo. This provides the opportunity to study compensatory mechanisms in developing nervous systems exposed to neurotoxicants, and to obtain repeated measures in the same organism over time, thereby reducing the number of animals required for testing. A major challenge confronting all *in vivo* systems-based DNT alternatives is determining the impact of potential species-specific toxicokinetic and toxicodynamic differences. How similar are xenobiotic metabolism and cellular defense mechanisms in these organisms to those of humans? However, the technology exists to “humanize” these organisms by stably expressing human genes (e.g., metabolic enzymes), so it may be possible to mitigate this problem.

#### Challenges to the development and application of DNT alternatives

Overall, a major challenge, which is driven by limited resources, is determining which of these *in vitro* cell-based and *in vivo* systems-based models to further develop as DNT alternatives. One approach is to evaluate each model against a set of characteristics defining the “ideal” DNT alternative. Although these defining characteristics require further discussion and refinement, it is widely accepted that the most important characteristic is that the model is predictive of neurotoxic responses in the developing human nervous system. Employing the strategy discussed above, alternative models for DNT would be based on conserved mechanisms of neurodevelopment. Additional characteristics of an ideal DNT alternative include sensitivity, specificity, and adaptability to high throughput/high content analyses. Other, more practical concerns are that the test be rapid, economical, and relatively simple to perform. These practical concerns are readily addressed for most candidate DNT alternatives, and there is considerable work ongoing to adapt many of these *in vitro* cell-based and *in vivo* systems-based models for high(er) throughput/high(er) content analyses. However, in general, there is insufficient data to rigorously evaluate the predictive validity, specificity or sensitivity of either *in vitro* cell-based or *in vivo* systems-based DNT alternatives. An immediate need, therefore, is to begin testing candidate models, ideally using a common set of mechanistically diverse developmental neurotoxicants to facilitate comparisons between models. Generating a reference set of chemicals to be used for testing alternative DNT models is another issue demanding immediate attention. An additional consideration is establishing the criteria for assembling a battery of alternative DNT models. For example, what types of data do regulators need, and what model characteristics are required to generate those data?

A second major challenge is coordinating the collection, storage, and analyses of DNT data. Ideally, all stakeholders would openly share information regarding alternative DNT model development and testing in a public database. Several issues will need to be addressed to achieve this goal, not least of which is identifying the resources to create and maintain a database. Also relevant to this challenge is determining how best to apply computational toxicology and systems biology to integrate structural and functional data with genomic, proteomic, and metabonomic data.

The resources and effort required to tackle these challenges are justified by the opportunity for these DNT alternatives to increase the rate of data acquisition while reducing the animal and other costs of DNT, thereby enhancing the ultimate goal of protecting children’s health. Data obtained from either *in vitro* cell-based or *in vivo* systems-based DNT alternatives may provide insights into relevant cellular, molecular, and regional targets, informing mode-of-action hypotheses and identifying potentially vulnerable developmental stages, thereby refining and reducing mammalian *in vivo* testing. A short-term goal is to identify DNT alternatives that can be used to prioritize chemicals for further testing, thereby focusing resources on those chemicals most likely to be hazardous to the developing nervous system. Long term, these alternative DNT models have the potential to replace at least some mammalian *in vivo* testing.

### Scientific and regulatory validation of alternative DNT models

The interface between developing science and its application to policy can be understood most effectively by discussing validation and regulatory acceptance. These concepts bridge the worlds of test development, laboratory research, and application of new test methods in regulation. A robust, timely, internationally harmonized, and effective path to validation and regulatory acceptance is essential for the successful deployment of alternatives in developmental neurotoxicology (Spielmann and Liebsch 2002).

#### Validation: the process

Validation is the process by which the reliability and relevance of a test are established for a particular purpose. In the context of validation, “reliability” refers to the inter- and intralaboratory reproducibility of a methodology [Garthoff 2005; Interagency Coordinating Committee for the Validation of Alternative Methods (ICCVAM) 1997; Zeigler and Stokes 1998] whereas “relevance” refers to a test’s ability to measure, predict, or model a toxicologic event of interest (Zeigler and Stokes 1998). In many cases, an alternative test is judged to be relevant if it produces information and data that are equivalent to or better than the information or data produced by a currently used *in vivo* test. Simply put, the benchmark for alternative tests is whether they are deemed to be as predictive as (or more predictive than) the tests they are intended to replace (Spielmann and Liebsch 2002).

Although the concept of validation is relatively easy to define and describe, in practice it remains challenging to implement for any single test. The process of validation is determined by a variety of factors including: *a*) the “comfort level” of the individual agencies or organizations that evaluate the test, which is a function of their statutory and regulatory mandate (Zeigler and Stokes 1998); *b*) influence from pressure groups (Garthoff 2005); and *c*) the perceived need for a new test protocol. Validation has evolved and improved in the past 15 years. Early lessons, arising out of the first unsuccessful attempts to validate alternative tests to replace the Draize eye procedure (Spielmann and Liebsch 2001), led to success in validating a number of tests, and has witnessed their acceptance by ICCVAM, ECVAM, and other international bodies, such as the OECD.

#### Validation: challenges and opportunities

Despite these successes, test validation remains challenging. First, it is very expensive (Spielmann 2003). Second, it is time consuming, taking 1.5–10 years (Garthoff 2005). Third, validation can require the use of large numbers of animals. Fourth, it can be difficult to find a sponsor or group of sponsors for the test (Spielmann and Liebsch 2002). Finally, as we discuss below, validation in and of itself offers no guarantee (at least in the United States) that the data generated by an alternative test will be accepted for use in decision making by a regulatory body (ICCVAM 1997; Zeigler and Stokes 1998). A specific challenge facing DNT alternatives is that predictive validity is harder to conceptualize in cases where correlative *in vivo* tests have not been established. Biostatistians and modelers can play an important role, and test developers should strongly consider developing predictive models based on statistics and systems biology (Spielmann and Liebsch 2002).

The presentations and discussions of the first TestSmart DNT workshop support the proposal that DNT alternatives, appropriately developed, can yield valuable data for decision making (Lein et al. 2005). The rate of throughput is another potential advantage of DNT alternatives over traditional *in vivo* tests. Alternatives that simultaneously test multiple end points of interest or multiple compounds would rapidly expand the knowledge base of developmental neurotoxicity. Successful use of these alternatives in decision making will require sorting them according to their long-, medium-, or short-term potential.

#### Regulatory acceptance: the process

Validation of an alternative test is a necessary but not the only step in using the test to make decisions that will affect regulations (Garthoff 2005). Once validated, a test must pass through another process to gain acceptance by regulatory agencies in the United States, the European Union, its member countries, and other jurisdictions. Regulatory acceptance is largely an ad hoc process that varies depending on *a*) the agencies and jurisdictions involved [e.g., the Centre for the Documentation and Evaluation of Alternative Methods to Animal Experiments (ZEBET) vs. OECD vs. U.S. EPA]; *b*) the particular law or regulation for which the testing data will be used; *c*) the influence of stakeholders, including business groups and advocacy groups for children’s health, animal welfare, and the environment; and *d*) the cultural norms of the jurisdiction that influence the ethics surrounding animal use in testing (Garthoff 2005; Schettler 2001; Spielmann 2003; Zeigler and Stokes 1998). In the United States, regulatory acceptance is determined by each individual agency. The statute that created the ICCVAM specifically separates validation and regulatory acceptance so that an agency may, at its discretion, accept or reject a validated test (ICCVAM Authorization Act 2000). It may also take an intermediate approach and use a validated test under one or more statutes for some purposes but not others (ICCVAM 1997). In the European Union, the journey from validation to regulatory acceptance appears to be less disjointed and more direct. Member countries are free to adopt additional procedures for regulatory acceptance, as are other international organizations or programs [OECD; Registration, Evaluation and Authorization of Chemicals (REACH); and Science, Children, Awareness, Legislation, Evaluation (SCALE)] (Garthoff 2005).

#### Regulatory acceptance: challenges and opportunities

To create incentives to develop alternatives and to maximize the regulatory use of the information generated by alternative tests will require that the gap between validation and regulatory acceptance be narrowed. A balance must be achieved between the needs of individual agencies to carry out their statutory mandates and the societal desire to implement the 3Rs (refining, reducing, and replacing the use of animals) while increasing testing of chemicals for developmental neurotoxic potential. DNT presents the opportunity to simultaneously achieve statutory directives and bring new alternatives on line, because as discussed during the TestSmart DNT Workshop, it is generally acknowledged that current DNT tests are not adequate for testing large numbers of chemicals. Nevertheless, the obstacles facing new tests in the regulatory area are substantial. To support alternatives, a more coordinated approach is needed. International harmonization presents a promising prospect for both simplifying and accelerating the pace of regulatory acceptance (Garthoff 2005; Spielmann 2003). A more integrated process of acceptance would serve the goals of accelerating the application of new knowledge, providing transparency for stakeholders and test developers, and protecting public health as called for by the statutory mandates that agencies must meet (Garthoff 2005).

## Workshop Conclusions and Recommendations

### Conclusions

The workshop achieved its primary objective to bring together the various stakeholders and individuals from diverse scientific disciplines to examine the TestSmart DNT goals and to share ideas and concerns relating to the science and policy of DNT. The goals of the TestSmart DNT program were generally supported across all stakeholder groups, and broad consensus was reached on the following conclusions:

1. There is a real and immediate need for an approach to DNT that incorporates faster and more efficient and cost-effective tests for developmental neurotoxicity. The DNT protocols currently approved for regulatory decision making are too expensive in terms of time, resources, and animals to meet the critical need of evaluating the thousands of chemicals for which there is currently little to no DNT information.
2. Short term, the goal is to identify DNT alternatives with predictive validity that could be used to prioritize chemicals for further testing, thereby focusing resources on exposures most likely to be hazardous to the developing human nervous system. The long-term goal is to develop policies that would incorporate DNT alternatives into regulatory decision making, which would significantly reduce, refine, and perhaps even replace current mammalian *in vivo* DNT protocols.
3. The overall strategy is to focus on DNT alternatives that assess chemical effects on evolutionarily conserved neurodevelopmental events. Coupling the tools and methods of cell and molecular developmental neurobiology with high throughput and/or high content technologies will be necessary to rapidly expand the DNT database. An immediate need is to initiate a systematic evaluation of alternative testing models to determine their relevance and reliability for DNT. Such an evaluation would be enhanced if DNT alternatives were tested against a generally accepted set of reference chemicals, which currently does not exist.
4. The creation of a high-quality open database to catalog existing DNT data and integrate new DNT data as they become available would significantly expedite the process of identifying and validating promising DNT end points and alternative DNT models. The usefulness of such a database would be enhanced significantly if it included access to DNT data generated by pharmaceutical, chemical, and other industries as part of their in-house product safety evaluations. Critical to the success of the database is identifying resources to support its creation and maintenance.
5. To achieve the TestSmart DNT goals it will be essential to continue to build a working group of scientists and policy makers who will maintain the momentum of the first workshop and interact to develop both the strategic goals and the process for establishing an alternative DNT testing system with broad applicability to protective public health decisions. Thus, as the science develops, its policy implications will be understood, and as policy needs are articulated, the science can respond.

### Recommendations

A pressing and immediate problem is the paucity of data regarding the predictive validity, specificity, and sensitivity of *in vitro* and alternative systems-based models for DNT. Such data are required not only to evaluate any given model, but also to determine how many end points and/or models are necessary for predictive validity. Thus, an immediate need is to begin testing candidate models—raising additional questions regarding which chemicals to use for these tests. Superimposed on these issues are the problems of limited resources (how do we prioritize models for testing?) and coordinating the collection, storage, and analyses of DNT data. Specific recommendations for questions to be addressed at the second TestSmart DNT workshop as they relate to each of the three overall goals of the TestSmart DNT program include:

#### Develop scientifically valid and efficient DNT alternatives

1. Develop a structured plan for DNT data collection and storage

- Develop a template for an open database that includes relevant fields for chemical- and end point–specific data
- Populate database with currently available DNT data and develop a structure for integrating evolving DNT data
- Provide open access for all stakeholders

1. Identify a reference set of chemicals for evaluating the reliability and relevance of alternative DNT models
2. Develop a decision framework for analyzing data generated using DNT alternatives

- Develop criteria for measuring success with DNT alternatives
- Integrate information from alternatives with data obtained from mammalian *in vivo* tests
- Apply computational toxicology and biostatistical approaches to evaluate the predictive validity of tests and test batteries

1. Identify resources to support development of DNT alternatives

#### Develop policies for incorporating DNT alternatives into regulatory decision making

1. Streamline the process of validation
2. Narrow the gap between validation and regulatory acceptance of DNT alternatives

- Develop transparent and scientifically robust criteria for the acceptance of validated tests in regulatory decision making
- Involve regulatory agencies throughout all stages of test development
- Review guidance and policies to ensure that these encourage application of data generated by alternatives
- Recognize the value of biologic endpoint data and its application to decision making

1. Harmonize validation and regulatory acceptance of DNT alternatives

- Within countries or jurisdictions sharing common regulatory organizations, harmonize validation and regulatory acceptance
- Between countries and jurisdictions, establish international principles for validation and regulatory acceptance
- Employ priority and tiered testing approaches that incorporate alternative testing protocols and data where feasible

#### Identify opportunities for reducing, refining, or replacing the use of animals in DNT

1. Refine current animal testing
2. Reduce the number of animals used in existing DNT protocols

- Apply data from DNT alternatives to focus testing in mammalian *in vivo* protocols to relevant end points
- Develop a tiered testing approach that can be used for regulatory decision making
- Ensure an international cooperative effort to streamline and harmonize the scientific validation of candidate DNT alternatives

1. Replace animals by validating alternative methods
